# *Piper nigrum* Leaf and Stem Assisted Green Synthesis of Silver Nanoparticles and Evaluation of Its Antibacterial Activity Against Agricultural Plant Pathogens

**DOI:** 10.1155/2014/829894

**Published:** 2014-01-15

**Authors:** Kanniah Paulkumar, Gnanadhas Gnanajobitha, Mahendran Vanaja, Shanmugam Rajeshkumar, Chelladurai Malarkodi, Kannaiyan Pandian, Gurusamy Annadurai

**Affiliations:** ^1^Environmental Nanotechnology Division, Sri Paramakalyani Centre for Environmental Sciences, Manonmaniam Sundaranar University, Alwarkurichi, Tamil Nadu 627412, India; ^2^Department of Inorganic Chemistry, University of Madras, Guindy, Chennai, Tamil Nadu 600 025, India

## Abstract

Utilization of biological materials in synthesis of nanoparticles is one of the hottest topics in modern nanoscience and nanotechnology. In the present investigation, the silver nanoparticles were synthesized by using the leaf and stem extract of *Piper nigrum*. The synthesized nanoparticle was characterized by UV-vis spectroscopy, X-ray diffraction (XRD), scanning electron microscope (SEM), transmission electron microscope (TEM), energy dispersive X-ray analysis (EDAX), and Fourier Transform Infrared Spectroscopy (FTIR). The observation of the peak at 460 nm in the UV-vis spectra for leaf- and stem-synthesized silver nanoparticles reveals the reduction of silver metal ions into silver nanoparticles. Further, XRD analysis has been carried out to confirm the crystalline nature of the synthesized silver nanoparticles. The TEM images show that the leaf- and stem-synthesized silver nanoparticles were within the size of about 7–50 nm and 9–30 nm, respectively. The FTIR analysis was performed to identify the possible functional groups involved in the synthesis of silver nanoparticles. Further, the antibacterial activity of the green-synthesized silver nanoparticles was examined against agricultural plant pathogens. The antibacterial property of silver nanoparticles is a beneficial application in the field of agricultural nanotechnology.

## 1. Introduction

Development of bioinspired material for fabrication of nanoparticle is the cutting edge of research in modern nanotechnology because of its ecofriendliness, involvement of nontoxic molecules, solvents, and suitable process for large-scale production [1]. Among the living organisms, the plant materials gain more attention in the nanoparticle production [2]. The heavy metal-resistant capability and phytoremediation of plants are the basic concept for the synthesis of nanoparticles. Haverkamp and Marshall [3] have demonstrated the uptake and conversion of metal salts like AgNO_3_, Na_3_Ag(S_2_O_3_)_2_, and Ag(NH_3_)_2_NO_3_ to metal silver nanoparticles when treated with *Brassica juncea*. Therefore, the metal-resistant capability of plants has motivated the researchers to develop environmentally benign novel green nanofactories for the synthesis of noble nanoparticles. Avoiding the maintenance of microbial cell culture is one of the advantages of plants when compared to the microbes. In addition, the plant-mediated synthesis is a rapid, flexible, and suitable process for large-scale production of nanoparticles. Nowadays, plant parts like fruit [4], leaf [2], bark [5], seed [6], and stem [7] extracts have been effectively used for synthesis of nanoparticles. Among nanoparticles, silver nanoparticles have been used enormously due to their potent antibacterial [8], antifungal [9], and antitumor activity [10]. Owing to the excellent antimicrobial properties, the silver nanoparticles have been widely used in food packaging [11], preservation [12], cosmetics [13], and medicine [14].

In the group of medicinal plants, the *Piper nigrum* possess excellent medicinal properties due to the presence of enormous phytochemicals. The piperine is an alkaloid, majorly found in *Piper nigrum*, which belongs to the Piperaceae family that is massively cultivated at India and Sri Lanka [15, 16]. Owing to the presence of large amount of phytochemicals, the leaf and stem of *Piper nigrum* are taken into account for the synthesis of silver nanoparticles. Further, the synthesized silver nanoparticles are characterized by UV-vis spectrophotometer, XRD, SEM, TEM, EDAX, and FTIR analysis. The antibacterial effect of silver nanoparticle is examined against plant pathogens such as *Citrobacter freundii* and *Erwinia cacticida*, which are isolated from *Abelmoschus esculentus* and *Citrullus lanatus*, respectively.

## 2. Materials and Methods

### 2.1. Preparation of *P. nigrum* Leaf and Stem Extracts

The *P. nigrum *leaves and stems are collected from the medicinal plant garden located in the MS University Campus at Alwarkurichi, India. The leaves are cut into small pieces and washed with the detergent tween 20 followed by double-distilled water for 2-3 times. It is slightly dried at room temperature. Individually, 10 g of leaf and stem is weighed and boiled with 100 mL of double-distilled water at 60–80°C for 10 min. After boiling, the solution is filtered through nylon mesh cloth and stored at 4°C for the nanoparticle synthesis.

### 2.2. Synthesis of Silver Nanoparticles by Using *P. nigrum* Leaf and Stem Extracts

For silver nanoparticle synthesis, about 10 mL of *P. nigrum *leaf and stem extract is added separately to 90 mL aqueous solution of AgNO_3_ (1 mM) (AgNO_3_ is purchased from Himedia Laboratories, India) and kept at room temperature. The color changes from pale yellow to brown indicating that the silver nanoparticles are formed as a result of the reaction of leaf and stem extracts of *P. nigrum *with silver metal ions. A control is maintained without addition of leaf and stem extract in the silver nitrate solution that shows no color changes.

### 2.3. Characterization Studies

The formation of silver nanoparticles is monitored by measuring the UV-vis spectra of the reaction mixture (Aqueous silver nitrate solution with *P. nigrum *leaf and stemextracts in separate conical flask). The UV-vis spectra measurements are carried out on Perkin-Elmer double-beam spectrophotometer operated with a resolution of 2 nm. Further, the reaction mixture is centrifuged at 10,000 rpm for 10 min and washed with double-distilled water. Repeat the centrifugation process for 4-5 times and allow the pellet to dry in hot air oven. The dried powder is used for further characterization studies. For XRD analysis, the powdered nanoparticles are coated on the amorphous silica substrate. The spectra are recorded by using XDL 3000 powder X-ray diffractometer with 40 kV and a current of 30 mA with Cu K*α* (1.5405 Å) radiation. The shape of the silver nanoparticles is examined by SEM experiments using Philips Scanning Electron Microscope. The presence of elemental silver is analyzed by energy dispersive spectroscopy attached to SEM. For TEM experiment, the silver nanoparticles are coated on copper grids and analyzed by Philips CM200 operated at 200 kV. The FTIR measurements are carried out using the Perkin-Elmer instrument at wavelength ranges from 4000 to 400 cm^−1^ at a resolution of 4 cm^−1^.

### 2.4. Isolation and Identification of Plant Pathogens

The *Citrobacter freundii* and *Erwinia cacticida* are isolated from *Abelmoschus esculentus* and *Citrullus lanatus*, respectively. The extract was collected from the disease infected leaf of the plant *Abelmoschus esculentus *and *Citrullus lanatus *and serially diluted the extract. Further, 0.1 ml of serially diluted extract from each dilution was spread on nutrient agar plates. The single colony was isolated and streak in a nutrient agar plates and incubated at 37°C for 24 hrs. The morphological and physiological characters of the isolated microorganism were performed based on the methods given in Bergys Manual of Determinative Bacteriology (Ed no. 9) [17].

### 2.5. Assessment of Antibacterial Activity of Green-Synthesized Silver Nanoparticles

Agar well diffusion method is used to examine the bactericidal effect of green-synthesized silver nanoparticles using the Muller-Hinton agar plates against the strains of *Citrobacter freundii* and* Erwinia cacticida*, which are isolated from the infected agricultural crops leaves such as *Abelmoschus esculentus* and *Citrullus lanatus*. The standard antibiotic (Chloramphenicol) is purchased from Himedia Laboratories, India. A single colony of test strain is grown overnight in LB broth medium on a rotary shaker (200 rpm) at 35°C. After 24 h of incubation, a loop full of bacterial culture is placed on the Muller-Hinton agar medium. Later, the silver nanoparticles (50 *μ*L), antibiotic (50 *μ*L), and silver nanoparticle (50 *μ*L) combined antibiotic solution are placed on the Muller-Hinton agar medium and incubated at 37°C for 24 h. After incubation, the zones of inhibition are measured.

## 3. Results and Discussion

### 3.1. Biochemical Identification of Plant Pathogens

**Figure 1:**
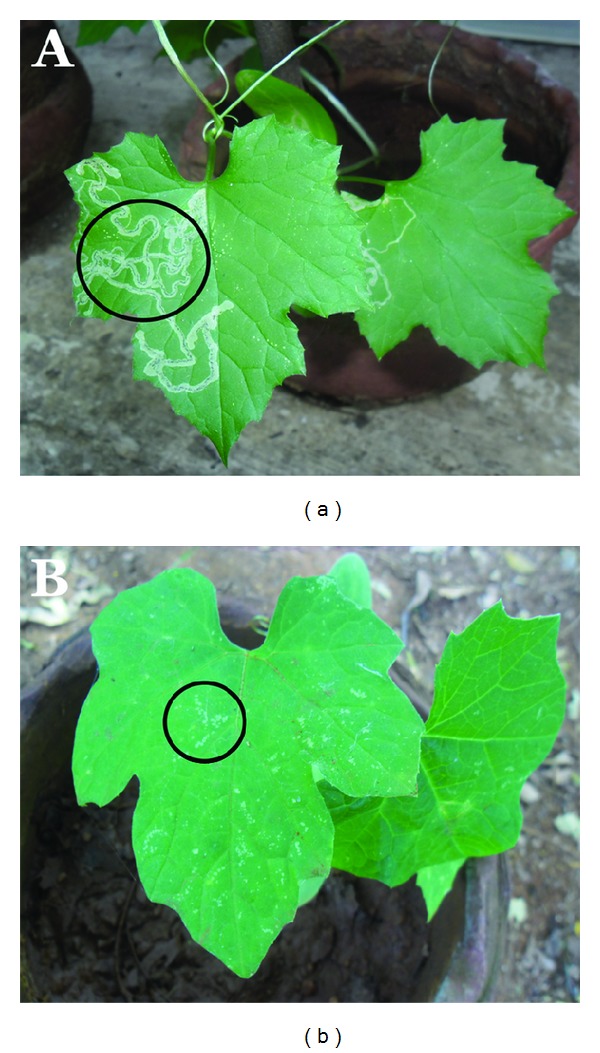
Diseases infected agricultural plant leaves of *Abelmoschus esculentus* and *Citrullus lanatus*.

The diseases infected agricultural plant leaves such as *Abelmoschus esculentus* and *Citrullus lanatus* are shown in Figures 1(a) and 1(b). The isolated microorganisms were tested by various cultural and biochemical tests for identification (Table 1). Gram's staining as preliminary identification to identify a newly isolated bacterium. This test gives a result, as gram positive, rod shape, and motile organism. The gram positive bacterium has the morphology as an abundant white waxy growth and highly motile. In addition, these bacteria do not have the ability to degrade the aminoacid tryptophan, so this test shows the negative result in indole test. The organism could not have the capacity to oxidize glucose with the production and stabilization of high concentration of acid end products, so this test gives negative result in methyl red. The organism does not have an ability to degrade urea by urease enzyme. So it gives negative result for urease test. This organism can degrade hydrogen peroxide by producing the enzyme catalase. In starch hydrolysis, these organisms have a hydrolytic activity and it also gives clear zone by addition of iodine solution. The above characteristics described that the above organism is found to be *Citrobacter freundii* and* Erwinia cacticida*.

### 3.2. Visual Identification

**Figure 2:**
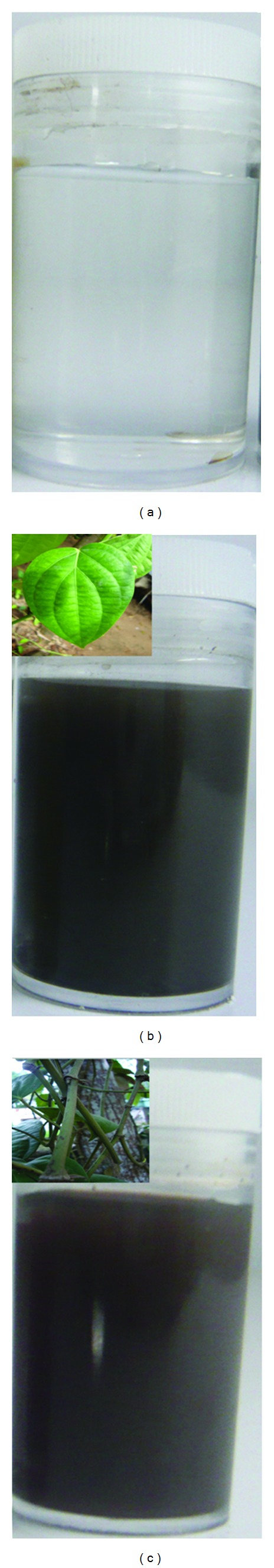
Visual observation of synthesis of silver nanoparticles before (a) and after addition of leaf (b) and stem (c) extract of *Piper nigrum* with silver nitrate solution. Inset of (b) and (c) shows *Piper nigrum* leaf and stem, respectively.

Biosynthesis of nanoparticles by using heterocyclic compounds gains more attention due to their simplicity and ecofriendly nature [18]. The medicinally important *P. nigrum *(leaf and stem) (Figures 2(b) and 2(c) inset) are taken into account and checked for their capability to synthesize the silver nanoparticles from silver nitrate solution. Recently, synthesis of nanoparticles using plants, particularly medicinal plant extracts, is gaining more attention due to the presence of active phytochemicals [4, 18–21]. The color identification is a preliminary analysis to confirm the formation of silver nanoparticles. In the present study, the formation of brown color indicates the synthesis of silver nanoparticles by using leaf and stem extracts of *P. nigrum* (Figures 2(b) and 2(c)).  Figure 2(a) shows that the silver nitrate solution without addition of *P. nigrum* leaf and stem extract can be treated as control. The color changes are acquired due to the excitation of SPR in the synthesized metal nanoparticles. Li et al. [19] and Ahmad et al. [21] reported that the *Capsicum annuum *and *Ocimum sanctum *extracts have taken 1 h to synthesize the silver nanoparticles. However, the report of Shankar et al. [18] shows that the silver nanoparticles are formed in 10 min in the solution of *Azadirachta indica*. The *P. nigrum *leaf and stem extracts show the formation of brown color in 10 min. This indicates that the silver nanoparticles synthesis process has been started. The intensity of brown color increases with increase in the duration of incubation.

### 3.3. UV-Vis Spectroscopic Analysis

**Figure 3:**
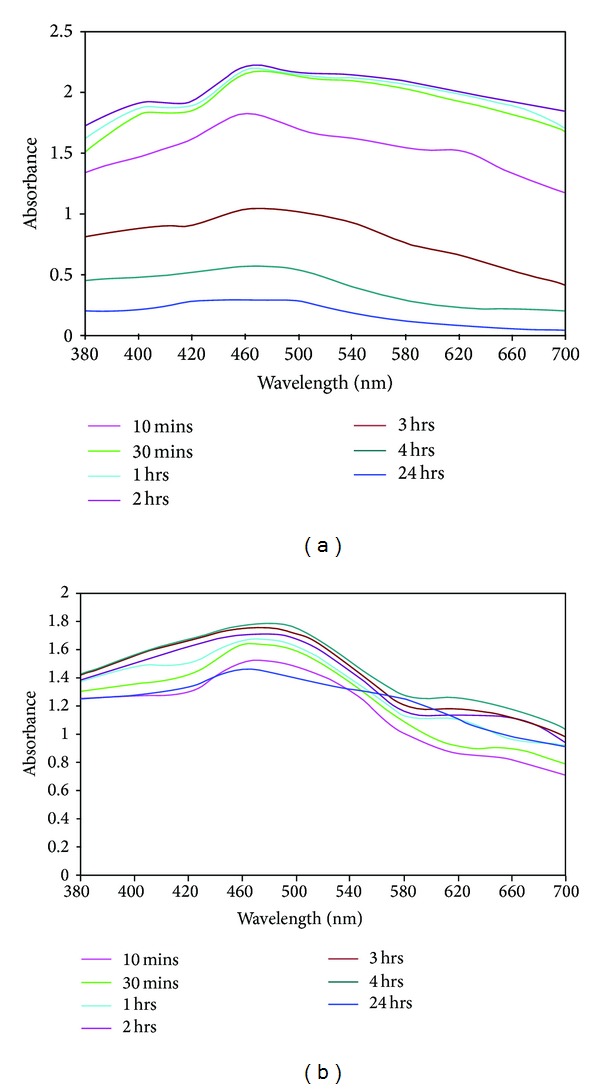
UV-vis spectra of *Piper nigrum* leaf- (a) and stem- (b) mediated synthesis of silver nanoparticles.

Figures 3(a) and 3(b) show the UV-vis spectra of the reaction mixture of silver nitrate solution with *P. nigrum *leaves and stem when exposed at different time intervals such as 10 min, 30 min, 1 h, 2 h, 3 h, 4 h, and 24 h. The peaks that are observed at 460 nm indicate the presence of silver nanoparticles that are synthesized by *P. nigrum *leaf and stem extracts. The peak is raised due to the effect of SPR on the synthesized metallic nanoparticles [22, 23]. The UV-vis spectrum of *P. nigrum *leaves signifies that the absorbance of silver nanoparticles is slowly increased from 10 min to 2 h. After 2 h, the low absorbance of nanoparticle indicates that the reaction has ended at 2 h (Figure 3(a)). After 2 h, the appearance of broad and low absorbance peak reveals the presence of large-sized nanoparticles due to the increased excitation of plasmon [24, 25]. The low absorbance of silver nanoparticles proves the presence of small and large spherical and irregular shaped nanoparticles with size ranging from 4 to 14 nm and 20 to 50 nm, respectively (Figures 7(a) and 7(b)). The TEM images of leaf-derived silver nanoparticles well agree with the report of UV-vis spectra. In leaf- and stem-mediated synthesis, the color intensity increased with increase in the duration of incubation. The nucleation of silver metal ions is acquired between 10 min and 2 h. Later, growth of silver nanoparticles is acquired, which leads to the development of small- and large-sized silver nanoparticles. In stem-mediated synthesis (Figure 3(b)), the 24 h absorption band (located close to 10 min) also confirms that the silver nanoparticles synthesis process was completed at 4 h incubation.

### 3.4. XRD Analysis

**Figure 4:**
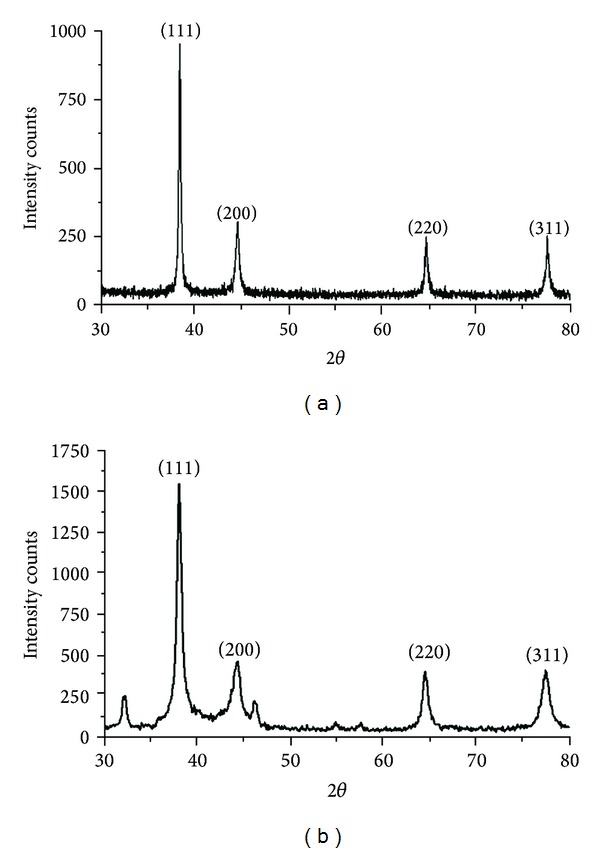
XRD analysis of green-synthesized silver nanoparticles by using leaf (a) and stem extract (b) of *Piper nigrum.*

The XRD spectra are used to confirm the crystalline nature of the silver nanoparticles synthesized by using *P. nigrum *(leaf and stem) and the patterns are exhibited in Figures 4(a) and 4(b). The spectra of XRD clearly indicate that the synthesized silver nanoparticles using the above-mentioned extracts are crystalline in nature. The Bragg reflections of silver nanoparticles are observed at 2*θ* values of 38.07°, 44.2°, 64.4°, and 77.5° for *P. nigrum *leaf and 38.09°, 46.1°, 67.3°, and 77.3° for *P. nigrum *stem. They correspond to the lattice planes (1 1 1), (2 0 0), (2 2 0), and (3 1 1), which were indexed for fcc silver. The obtained Bragg peaks are compared with pure crystalline silver published by Joint Committee on Powder Diffraction Standards (File no. 04-0783). The average sizes of the silver nanoparticle synthesized by leaf and stem extract of *P. nigrum* are estimated from the broadening plane (1 1 1) using the Debye Scherrer equation

(1) $$\begin{array}{c} D=\frac{K\lambda }{\beta \cos\theta }, \end{array}$$

where *D* is the average particle size, *k* is the shape factor (constant 0.9), *λ* is the X-ray wavelength (1.5406 Å), *β* is the full width at half maximum of the peak (FWHM), and *θ* is the diffraction angle. The average sizes of the silver nanoparticle synthesized by leaf and stem extract of *P. nigrum* are around 27 nm and 13.5 nm, respectively, which is well matched with the result of TEM analysis.

### 3.5. SEM and EDAX Analysis

**Figure 5:**
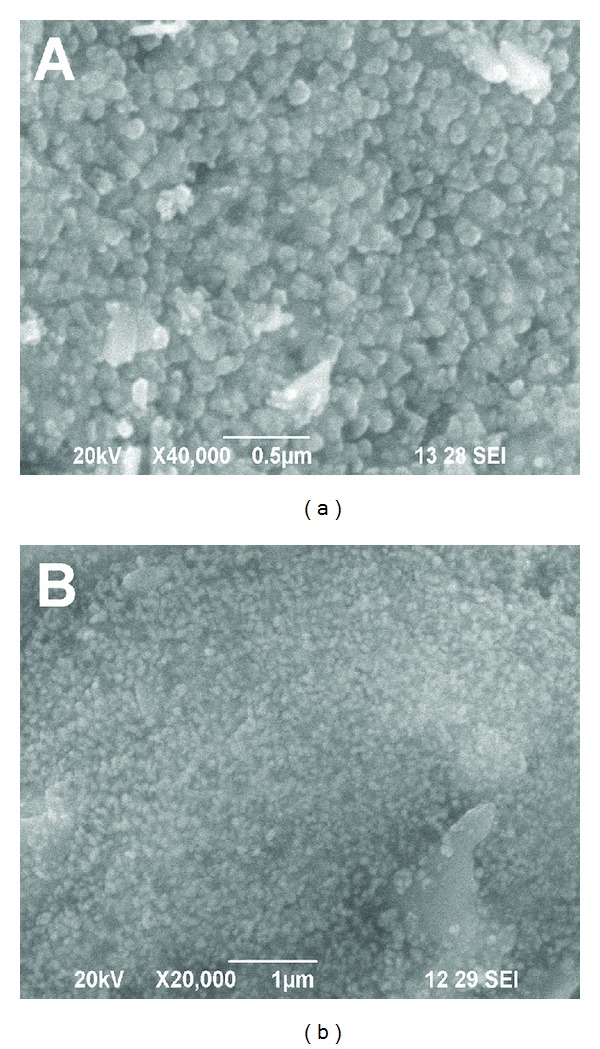
SEM images of *P. nigrum* leaf- (a) and stem- (b) synthesized silver nanoparticles.

**Figure 6:**
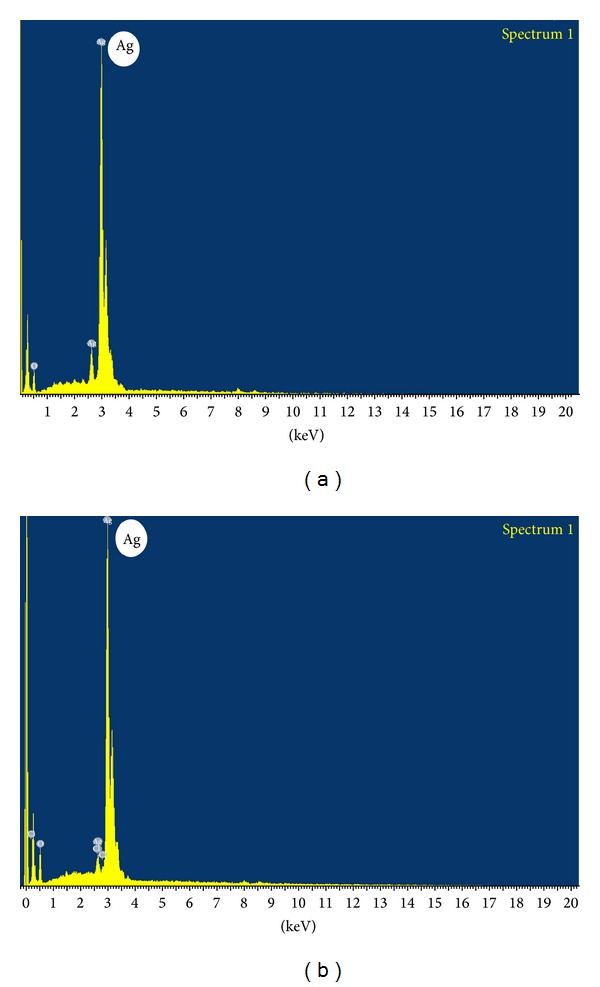
EDAX spectra of silver nanoparticles synthesized by using leaf (a) and stem extract (b) of *Piper nigrum.*

Scanning electron microscope is one of the powerful tools to identify the shape of the nanoparticles. The silver nanoparticles synthesized by the *P. nigrum *(leaf and stem) are predominantly spherical in shape (Figures 5(a) and 5(b)) (Scale bar 500 nm). The aggregation of nanoparticles is acquired only after 2 h and 4 h for *P. nigrum *leaf and stem extract, respectively, resulting in the formation of large-sized nanoparticles. In *P. nigrum *leaf, the reduction of silver metal ions into silver nanoparticles is acquired from 5 min to 2 h. After 2 h, the growth of nanoparticles leads to the formation of small and large-sized spherical and irregular shaped silver nanoparticles. Further, elemental analysis is carried out to confirm the presence of metallic silver nanoparticles in the reaction mixture. Figures 6(a) and 6(b) show the EDAX analysis of *P. nigrum *(leaf and stem)-mediated synthesis of silver nanoparticles. The EDAX analysis shows an intense signal at 3 keV indicating that the presence of elemental silver is biofabricated by the *P. nigrum *leaf and stem extracts.

### 3.6. TEM Analysis

**Figure 7:**
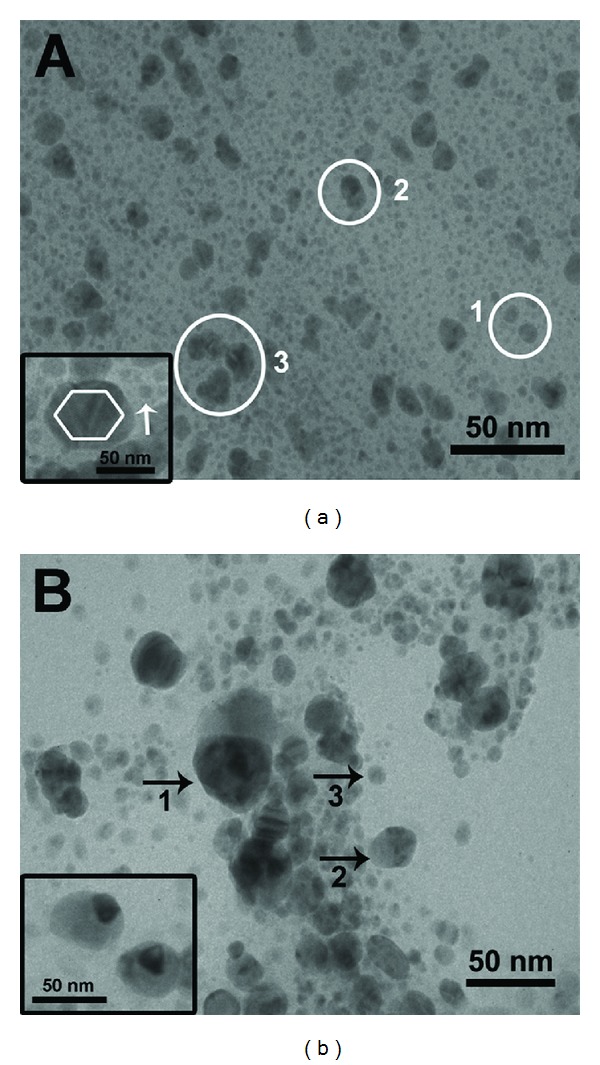
TEM images of silver nanoparticles synthesized by using leaf (a) and stem extract (b) of *Piper nigrum.*

The TEM images of *P. nigrum *(leaf and stem) extracts synthesized silver nanoparticles are shown in Figures 7(a) and 7(b) and the images signify that the synthesized silver nanoparticles are polydispersed. Entirely, the synthesized silver nanoparticles are spherical in shape and some undefined shapes are also observed with slight aggregation (Figures 7(a) and 7(b)). The *P. nigrum *leaf-synthesized silver nanoparticles vary from 4–14 nm for small-sized nanoparticles (marked as circles 1 and 2 in Figure 7(a)) to 20–50 nm for large-sized nanoparticles (marked as circle 3 in Figure 7(a)). Additionally, hexagonal and small spherical nanoparticles (arrow marked in Figure 7(a) inset) are also observed (Figure 7(a) inset). Interestingly, the twined nanoparticles were found (Figure 7(b) inset), which are synthesized by using the stem extract of *P. nigrum*. Similarly, Wiley et al. [26] obtained the twined silver nanoparticles synthesized by ethylene glycol in the presence of poly (vinyl pyrrolidone) and a trace amount of sodium chloride. Here, the size of the obtained silver nanoparticles synthesized by stem extracts of *P. nigrum* is in the range 9–30 nm (Figure 7(b)).

### 3.7. FTIR Analysis

**Figure 8:**
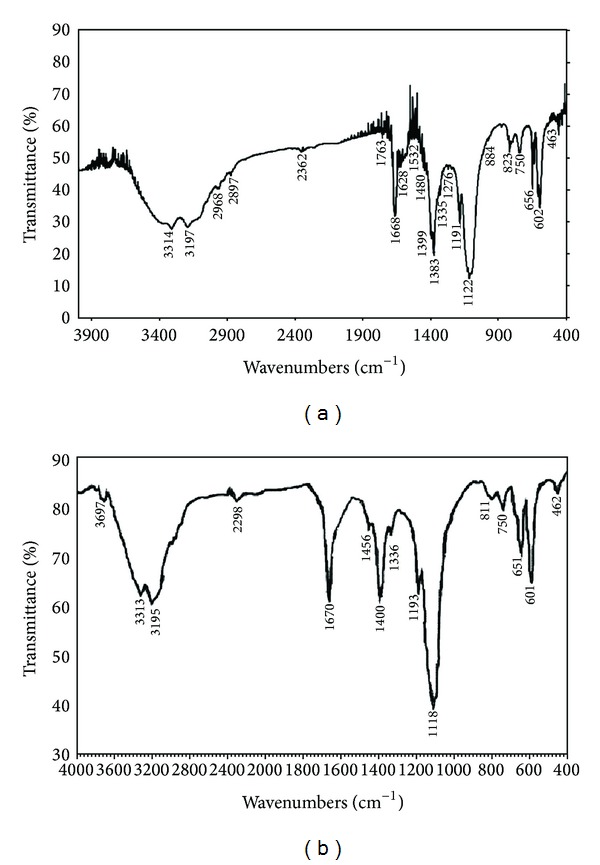
FTIR spectra of silver nanoparticles synthesized by using leaf (a) and stem extract (b) of *Piper nigrum.*

The possible functional groups of phytochemicals in plant extract involved in nanoparticles synthesis are identified by FTIR analysis. The silver nanoparticles synthesized by leaf extract of *P. nigrum *(Figure 8(a)) exhibit intense absorption peaks at 3314 cm^−1^ and 3197 cm^−1^ corresponding to N–H stretching of primary amine. The weak band observed at 2897 cm^−1^ and 2362 cm^−1^ indicates the H–C–H asymmetric and symmetric stretching of alkanes. The band observed at 2362 cm^−1^ denotes the presence hydrogen-bonded OH stretching of carboxylic acids in the leaf extract, which may be a reducing agent responsible for the synthesis of silver nanoparticles. Dubey et al. [27] reported that the carboxylate ions of sorbic acid in *Sorbus aucuparia *are responsible for the development of silver and gold nanoparticles. The absorption band at 1763 cm^−1^, 1668 cm^−1^, and 1628 cm^−1^ represents the C=O stretching of ketones. The peaks observed at 1532 cm^−1^ and 1480 cm^−1^ denote the N–H bending of secondary amine and the band at 1399 cm^−1^, 1383 cm^−1^, and 1335 cm^−1^ exemplifies the N=O stretching of nitro groups of leaf extract. The arising of these functional groups in FTIR spectrum indicates the association of silver nanoparticles with the phytochemicals. The absorption band at 1276 cm^−1^, 1191 cm^−1^, and 1122 cm^−1^ indicates the C–O stretching of esters and the band at 884 cm^−1^, 823 cm^−1^, 750 cm^−1^, 656 cm^−1^, and 602 cm^−1^ represents the C–H bending of alkynes. In stem-derived silver nanoparticles (Figure 8(b)), the band observed at 3697 cm^−1^ represents the presence of primary alcohol and the absorption of intense band at 3313 cm^−1^ and 3195 cm^−1^ denotes the N–H stretching of primary amine. The band at 2298 cm^−1^ corresponds to nitrile groups. The peak at 1456 cm^−1^ and 1336 cm^−1^ denotes the N–H bending of secondary amines. The strong band observed at 1670 cm^−1^ denotes the presence of C=O stretching of amides of the stem extracts. The phytochemicals of *P. nigrum* stem extracts are bound to the silver nanoparticles through amide and amine groups. The weak band at 1193 cm^−1^ and 1118 cm^−1^ denotes the C–O stretching of esters and the band observed at 811 cm^−1^, 750 cm^−1^, 651 cm^−1^, and 601 cm^−1^ corresponds to C–H bending of alkynes.

### 3.8. Antibacterial Activity of Green-Synthesized Silver Nanoparticles

**Figure 9:**
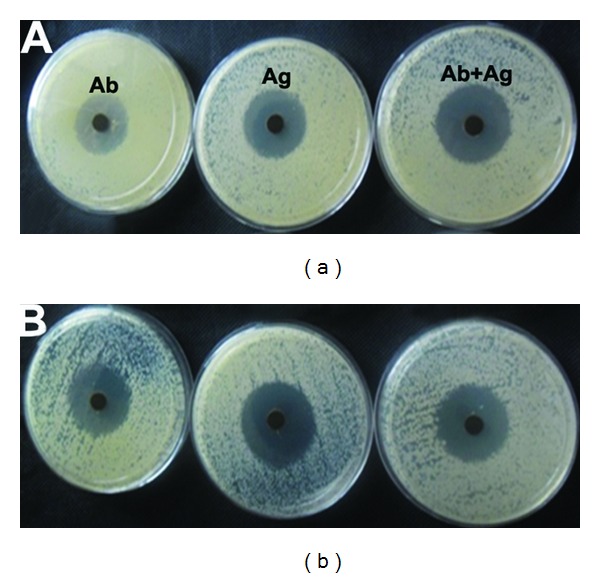
Antibacterial activity of silver nanoparticles synthesized by using leaf and stem extract of *Piper nigrum.*

The well diffusion method was carried out to examine the antibacterial activity of green synthesized silver nanoparticles under in vitro conditions against *Citrobacter freundii *and *Erwinia cacticida *(Figure 9). The silver nanoparticle (leaf synthesized) combined antibiotic solution exhibits admirable zone of inhibition at 50 *μ*L against *Citrobacter freundii* and *Erwinia cacticida* (15.853 ± 0.153 and 16.295 ± 0.203, resp.) (Table 2). Similarly, the silver nanoparticle (stem- synthesized) combined antibiotic solution also shows maximum zone of inhibition of 12.833 ± 0.441 and 14.533 ± 0.291 against *Citrobacter freundii* and *Erwinia cacticida*, respectively (Table 2). The silver nanoparticle (leaf- and stem-synthesized) combined antibiotic solution shows fine zone of inhibition when compared to the antibiotic and silver nanoparticles. There is a slightly increased zone of inhibition that is observed in leaf-synthesized silver nanoparticles when compared to the stem-synthesized silver nanoparticles. The results clearly demonstrate that newly synthesized silver nanoparticles are promising antimicrobial agents against the plant pathogens employed. When compared to silver nanoparticles, the silver nanoparticle combined antibiotic solution shows enhanced zone of inhibition against the plant pathogens. The green-synthesized silver nanoparticle is a good source, which is easily produced and extensively useful in biomedical and agricultural applications.

The exact mechanism of antibacterial activity of silver nanoparticles is still unknown. Some of the researchers have demonstrated the plausible mechanism of antimicrobial effect of silver nanoparticles. One of the reasons is that the formation of free radicals produced from the nanoparticles could disturb the membrane lipids and then finally spoil the membrane functions [28, 29]. Danilczuk et al. [28] and Kim et al. [29] have confirmed the formation of free radicals and membrane damage is confirmed by the most useful ESR studies. However, Stoimenov et al. [30] and Sondi and Salopek-Sondi [31] have depicted a new finding that the membrane could be damaged by the formation of pits on the surface of the bacterial cell wall membrane. The formation of pits on the membrane leads to increase in the permeability and irregular transport that result in the death of the bacterial cells. This concept is also illustrated by Amro et al. [32] that the depletion of metal particles might form irregular shaped pits on the outer membrane of the bacteria and result in the damage of membrane permeability. The membrane interruption leads to the release of lipopolysaccharides and finally the cell is totally collapsed.

## 4. Conclusion

The present investigation demonstrates the green-synthesis of silver nanoparticles by using the leaf and stem extracts of *P. nigrum*. In both, the synthesis of silver nanoparticles using leaf and stem extracts of *P. nigrum* is started at 10 min and ended at 2 h for leaf and 4 h for stem extracts. Interestingly, the TEM images stem-synthesized silver nanoparticles show twine-shaped silver nanoparticles. The size of the stem-derived silver nanoparticles is 9–30 nm, whereas, the small- and large-sized silver nanoparticles are observed in TEM images of leaf-synthesized silver nanoparticles and the size ranges from 4 to 14 nm for small-sized nanoparticles and 20–50 nm for large-sized nanoparticles. The leaf- and stem-synthesized silver nanoparticles have shown excellent antimicrobial activity against agricultural plant pathogens *Citrobacter freundii* and *Erwinia cacticida*. The silver nanoparticle-impregnated antibiotic disc shows fine zone of inhibition when compared to antibiotic and silver nanoparticles. Thereby, the results of the present study conclude that the silver nanoparticles were synthesized by using the cheapest and ecofriendly leaf and stem extracts of *P. nigrum*. The excellent antibacterial activity of silver nanoparticles against plant pathogens will have a beneficial application in crop improvement and protection in agricultural nanotechnology.

## Figures and Tables

**Table 1:** Biochemical analysis of bacteria isolated from disease infected agricultural plant leaf.

S. no.	Biochemical tests	*Citrobacter freundii *	*Erwinia cacticida *
1	Gram staining	Gram negative Rod	Gram negative rods
2	Agar slant	Small white rounded, waxy colonies	Translucent ivory and convex with entire margins
3	Spore staining	Negative	Negative
4	Indole	Negative	Positive
5	Methyl red	Positive	Negative
6	Voges Proskauer	Negative	Positive
7	Citrate utilization	Positive	Positive
8	Starch hydrolysis	Negative	Negative
9	Oxidase	Negative	Negative
10	Catalase	Positive	Positive
11	Urease activity	Variable	Negative
12	Triple sugar iron agar test	Acid and H_2_S production	Acid production
13	H_2_S production	Positive	Negative

**Table 2:** Evaluation of antibacterial activity of silver nanoparticles synthesized by using leaf and stem extract of *Piper nigrum*.

S. no.	Name of the bacterial strain	*Piper nigrum* leaf (zone of inhibition in mm)			*Piper nigrum* stem (zone of inhibition in mm)	
		Ab	Ag (50 *µ*L)	Ag + Ab (50 *µ*L)	Ag (50 *µ*L)	Ag + Ab (50 *µ*L)
1	*Citrobacter freundii *	8.023 ± 0.176	8.962 ± 0.203	15.853 ± 0.153	8.894 ± 0.120	12.833 ± 0.441
2	*Erwinia cacticida *	8.643 ± 0.195	9.052 ± 0.153	16.295 ± 0.203	9.012 ± 0.245	14.533 ± 0.291

Ab: antibiotic; Ag: silver; Ag + Ab: silver nanoparticles combined antibiotic solution.
